# No effect of 25-hydroxyvitamin D supplementation on the skeletal muscle transcriptome in vitamin D deficient frail older adults

**DOI:** 10.1186/s12877-019-1156-5

**Published:** 2019-05-28

**Authors:** Roland W. J. Hangelbroek, Anouk M. M. Vaes, Mark V. Boekschoten, Lex B. Verdijk, Guido J. E. J. Hooiveld, Luc J. C. van Loon, Lisette C. P. G. M. de Groot, Sander Kersten

**Affiliations:** 1 Top Institute Food and Nutrition, P.O. Box 557, 6700 AN Wageningen, The Netherlands; 2 Division of Human Nutrition and Health, Wageningen University, Stippeneng 4, 6708 WE Wageningen, The Netherlands; 3 Department of Human Biology and Movement Sciences, NUTRIM School for Nutrition and Translational Research in Metabolism, Maastricht University Medical Centre, P.O. Box 616, 6200 MD Maastricht, The Netherlands

**Keywords:** Vitamin D, 25-hydroxyvitamin D, older adults, transcriptomics, skeletal muscle

## Abstract

**Objective:**

Vitamin D deficiency is common among older adults and has been linked to muscle weakness. Vitamin D supplementation has been proposed as a strategy to improve muscle function in older adults. The aim of this study was to investigate the effect of calcifediol (25-hydroxycholecalciferol) on whole genome gene expression in skeletal muscle of vitamin D deficient frail older adults.

**Methods:**

A double-blind placebo-controlled trial was conducted in vitamin D deficient frail older adults (aged above 65), characterized by blood 25-hydroxycholecalciferol concentrations between 20 and 50 nmol/L. Subjects were randomized across the placebo group and the calcifediol group (10 μg per day). Muscle biopsies were obtained before and after 6 months of calcifediol (*n* = 10) or placebo (*n* = 12) supplementation and subjected to whole genome gene expression profiling using Affymetrix HuGene 2.1ST arrays.

**Results:**

Expression of the vitamin D receptor gene was virtually undetectable in human skeletal muscle biopsies, with Ct values exceeding 30. Blood 25-hydroxycholecalciferol levels were significantly higher after calcifediol supplementation (87.3 ± 20.6 nmol/L) than after placebo (43.8 ± 14.1 nmol/L). No significant difference between treatment groups was observed on strength outcomes. The whole transcriptome effects of calcifediol and placebo were very weak, as indicated by the fact that correcting for multiple testing using false discovery rate did not yield any differentially expressed genes using any reasonable cut-offs (all q-values ~ 1). *P*-values were uniformly distributed across all genes, suggesting that low *p*-values are likely to be false positives. Partial least squares-discriminant analysis and principle component analysis was unable to separate treatment groups.

**Conclusion:**

Calcifediol supplementation did not significantly affect the skeletal muscle transcriptome in frail older adults. Our findings indicate that vitamin D supplementation has no effects on skeletal muscle gene expression, suggesting that skeletal muscle may not be a direct target of vitamin D in older adults.

**Trial registration:**

This study was registered at clinicaltrials.gov as NCT02349282 on January 28, 2015.

## Background

Muscle weakness and muscle loss increase with age, potentially leading to increased risk of falls, frailty and loss of independence among older adults [1]. One of the factors that may influence muscle health is vitamin D. Vitamin D is partly obtained from the diet and is produced endogenously in the skin via a photochemical reaction. Vitamin D is mainly known for its role in bone health by promoting the absorption of calcium in the intestine and the retention of calcium in the kidneys. Many older adults are deficient or insufficient in vitamin D [2, 3]. In a recent study in 4495 European individuals aged > 65 years, vitamin D deficiency (serum 25-hydroxyvitamin D 25–50 nmol/L) and vitamin D insufficiency (50–75 nmol/L) were found in 41 and 33% of the population, respectively [3]. Various observational studies have found associations between vitamin D deficiency and impaired muscle function and/or physical performance in older adults [4–8]. As a consequence, vitamin D supplementation has been proposed as a strategy to improve muscle function [9].

Vitamin D is suspected to affect muscle health via both indirect and direct mechanisms. Indirectly vitamin D can influence muscle function via its role in calcium and phosphate homeostasis [10, 11]. Vitamin D has also been proposed as a direct modulator of skeletal muscle signalling via activation of the vitamin D receptor (VDR). Several cell culture studies have suggested a role of VDR signalling in skeletal muscle function [12–15]. In addition, inactivation of the VDR in mice leads to impaired muscle development and differential expression of key myogenic regulators [16]. Conversely, calcitriol (1,25-dihydroxyvitamin D3) was found to inhibit myoblast proliferation and differentiation in primary myocytes isolated from human skeletal muscle [17].

While in vitro and animal studies thus support a role for VDR in gene regulation in muscle cells [18], more recently the function of VDR in skeletal muscle has come into question [19]. Wang and colleagues showed that most of the antibodies directed against VDR lacked specificity, potentially leading to false positives [20]. Intriguingly, despite the fact that VDR was present and functional in myocytes isolated from human skeletal muscle and C2C12 myotubes, Olsson and colleagues were unable to detect appreciable levels of VDR in mature human skeletal muscle [17]. Here we aimed to determine the overall impact of vitamin D on skeletal muscle gene regulation in vivo in humans and identify potential VDR target genes in human skeletal muscle. To that end, we conducted a transcriptomics analysis on muscle biopsies obtained from frail older adults participating in a randomized, placebo-controlled double blind trial investigating the effect of calcifediol (25-hydroxycholecalciferol or 25(OH)D) supplementation on muscle function [21].

## Methods

### Study Design & Population

This study is part of a larger clinical trial that studied the effect of calcifediol (25-hydroxyvitamin D_3_ or 25(OH)D_3_) and cholecalciferol (vitamin D_3_) on muscle strength. Procedures for this study have been described elsewhere [21]. This study used a randomized, parallel- arm double blind design. All subjects had a serum 25-hydroxycholecalciferol concentration between 20 and 50 nmol/L. A serum 25-hydroxycholecalciferol concentration of 50 nmol/l (20 ng/ml) is often used as a threshold for vitamin D deficiency and for vitamin D supplementation [22]. Subjects in the calcifediol arm received 10 μg (400 IU) 25-hydroxycholecalciferol per day (DSM Nutritional Products Ltd.). Subjects were instructed to take their capsules in the morning during breakfast. Treatment compliance was reported at 3 and 6 months by capsule count of returned capsules, taking into account the number of days active in the study. Participants were considered compliant when ≥80% of the study supplements were taken. Overall compliance to treatment was ≥80% in all participants, with an average compliance of 98%. Participants were frail and pre-frail older adults (65+) with serum levels of 25-hydroxyvitamin D3 between 20 and 50 nmol/L. Frailty was assessed using the Fried criteria [23]. Power analysis for the larger clinical trial was performed using knee-extension strength as a primary outcome measure [21]. A subset of samples was taken from the main study based on how much muscle was available for transcriptomics analysis (12 subjects in the placebo arm and 10 subjects in the calcifediol arm). No power analysis was performed to determine the number of subjects needed for the transcriptomics analysis. The study was approved by the Medical Ethics Committee of Wageningen University. All participants gave their written informed consent. The study was registered at clinicaltrials.gov as NCT02349282.

### Strength measurements

Isometric leg muscle strength (leg extension and leg flexion) was assessed using a Biodex System 4 dynamometer (Biodex Medical Systems, Shirley, NY, USA). Subjects were seated upright with their chest and waist secured by belts. Experiments were performed with knee angle of 60^0^ and hip angle of 90^0^. Subjects performed 3 maximal voluntary isometric contractions for five seconds, with 30 s of rest between trials and five minutes of rest between knee-extension and knee-flexion trials. Researchers provided standardized verbal encouragement during the strength tests.

### Blood samples

Blood samples were collected in a fasting state in the morning and stored at − 80 °C until analysis. Serum 25(OH)D_3_ (nmol/L) and 24,25(OH_2_)D_3_ (nmol/L) were analyzed using LC/MS/MS (Analytical Research Center, DSM Nutritional Products, Kaiseraugst, Switzerland) as previously described [21, 24].

### Muscle biopsies

Muscle biopsies were collected at baseline and after 6 months of supplementation. The last dose of supplement was taken the preceding day. Subjects were in the fasted state when the biopsy was taken. Muscle biopsies were taken from the middle region of the *vastus lateralis* muscle under local anaesthesia, ~ 15 cm above the patella and ~ 3 cm below entry through the fascia, using the percutaneous needle biopsy technique [25]. Muscle samples were dissected carefully and freed from any visible non-muscle material and were immediately frozen in liquid nitrogen. Subsequently, muscle samples were stored at − 80 °C until further analysis.

### Microarray analysis and qPCR

RNA was isolated (RNeasy Micro kit, Qiagen, Venlo, the Netherlands), quantified (Nanodrop ND 1000, Nanodrop technologies, Wilmington, DE, USA) and integrity was checked by an Agilent 2100 Bioanalyser with RNA 6000 microchips (Agilent Technologies, South Queensferry, UK). Total RNA was labelled using the GeneChip® WT plus Reagent Kit and hybridized to GeneChip® Human Gene 2.1 ST Array (Affymetrix, Inc. Santa Clara, CA, USA). Sample labelling, hybridization to chips, and image scanning were performed according to the manufacturers’ instructions.

We performed qPCR on the VDR gene using gene- specific primers (forward: GTGGACATCGGCATGATGAAG, reverse: GGTCGTAGGTCTTATGGTGGG). 500 ng RNA was reverse transcribed to cDNA using a iScript cDNA synthesis kit (Bio-Rad Laboratories, Veenendaal, Netherlands). Real-time PCR was performed using SensiMix (Bioline, GC biotech, Alphen aan den Rijn, Netherlands) on a CFX384 Real-Time PCR detection system (Bio-Rad Laboratories, Veenendaal, Netherlands).

### Data analysis

All data analysis was done in *R* [26]. Changes in muscle strength and vitamin D levels were evaluated using linear mixed models using the *lme4* library [27]. Microarray data were assessed for quality using the MADMAX pipeline and additionally by visually inspecting the probe level residuals and NUSE (Normalized Unscaled Standard Error) plots [28]. Data was normalized using Robust Multichip Average (RMA) [29]. Gene level summarization was performed using version 22 of the Custom CDF from the Brainarray project [30]. Genes were filtered using Universal ExPression Codes (UPC) filtering with a 50% expression likelihood cut-off in at least 10 samples, the smallest subset within this dataset [31].

Univariate statistical analysis of gene expression was performed using the limma R library [32]. Contrasts were set for time effect in both placebo and calcifediol groups and an interaction term for the calcifediol group versus the placebo group. *P*-values were calculated using Intensity Based Moderated t-tests [33]. Genes with a *p*-value below 0.05 and an absolute fold change above 1.2 were considered statistically significant. Gene ontology was performed using EnrichR [34]. Pathway and upstream regulator analysis was performed using Ingenuity Pathway Analysis (Qiagen, The Netherlands). Partial least squares discriminant analysis (PLS-DA) was performed using the *caret* and *pls* libraries [35]. PLS-DA model was validated using 5 × 5-fold repeated cross-validation. Model performance was evaluated using area under ROC curve (AUROC). Receiver Operator Characteristic (ROC) curve and heatmaps were made using *ROCR* and *ComplexHeatmap* libraries, respectively [36, 37].

## Results

### Vitamin D status and muscle strength

Calcifediol supplementation led to significant increases in total 25(OH)D_3_ and 24,25(OH_2_)D_3_ levels compared to placebo (Table 1). At the end of the study, subjects in the placebo group were on average still below the deficiency cut-off used for this study (50 nmol/L), whereas the calcifediol group was not. No differences were observed in muscle strength outcomes (BioDex leg extension and flexion peak torque, Table 1). A full discussion of physiological outcomes of calcifediol supplementation can be found elsewhere [21].

**Table 1:** Subject characteristics and main effect of calcifediol supplementation

	Placebo - Pre	Placebo - Post	25(OH)D - Pre	25(OH)D - Post
N	12		10	
Gender (M / F)	6 / 6		6 / 4	
Age (years)	74.1 ± 5.8		71.8 ± 5.7	
BMI (kg/m^2^)	27.2 ± 4.0		28.0 ± 4.0	
Weight (kg)	76.2 ± 14.0		80.7 ± 14.9	
Body Fat (%)	32.1 ± 6.9	31.3 ± 6.6	32.5 ± 7.8	32.4 ± 7.8
Total 25(OH)D_3_ (nmol / L)	37.5 ± 11.9	43.8 ± 14.1	34.1 ± 9.3	87.3 ± 20.6
24,25(OH_2_)D_3_ (nmol / L)	1.9 ± 0.8	2.7 ± 1.5	1.8 ± 0.7	8.0 ± 3.7
BioDex Leg Extension Peak Torque (Nm)	129.7 ± 48.9	133.6 ± 64.1	145.9 ± 50.3	157.2 ± 56.1
BioDex Leg Flexion Peak Torque (Nm)	57.0 ± 29.9	55.2 ± 35.0	64.8 ± 22.5	70.0 ± 23.6

Values are means ± SDs

### Effect of Calcifediol on muscle transcriptome

**Fig. 1:**
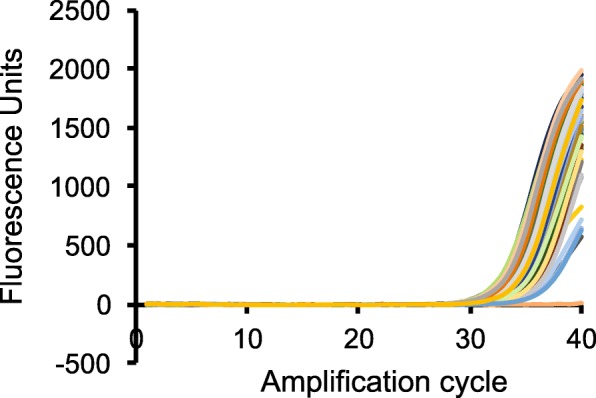
Very low expression of VDR in human skeletal muscle. qPCR Amplification curves of VDR in muscle biopsies. cDNA obtained from human muscle biopsies was PCR amplified using primers against human VDR. The different lines represent different subjects

The muscle biopsies were used for qPCR analysis and transcriptomics. The Ct values for qPCR amplification of VDR in the muscle biopsies were above 30 (Fig. 1), suggesting that VDR is expressed at very low levels in human skeletal muscle. Microarray analysis confirmed the very low VDR mRNA expression in the human muscle biopsies, with a median raw intensity of 12.

**Fig. 2:**
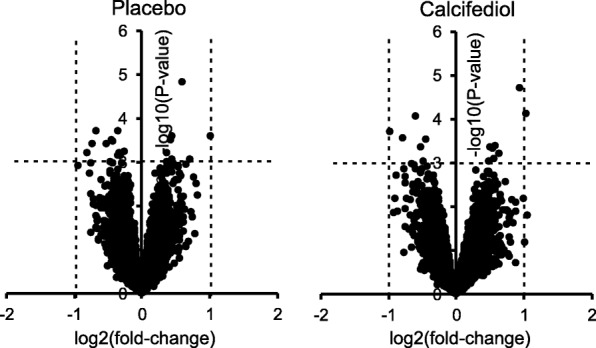
Minimal effect of calcifediol and placebo on skeletal muscle gene expression in vitamin D deficient frail older adults. Volcano plot showing the relation between signal log ratio (log2[fold-change], x-axis) and the -log10 of the IBMT *P*-value (y-axis) for the effect of calcifediol and placebo on the skeletal muscle transcriptome. The dotted lines show the threshold for fold-change of 2, and P-value of 0.001

**Fig. 3:**
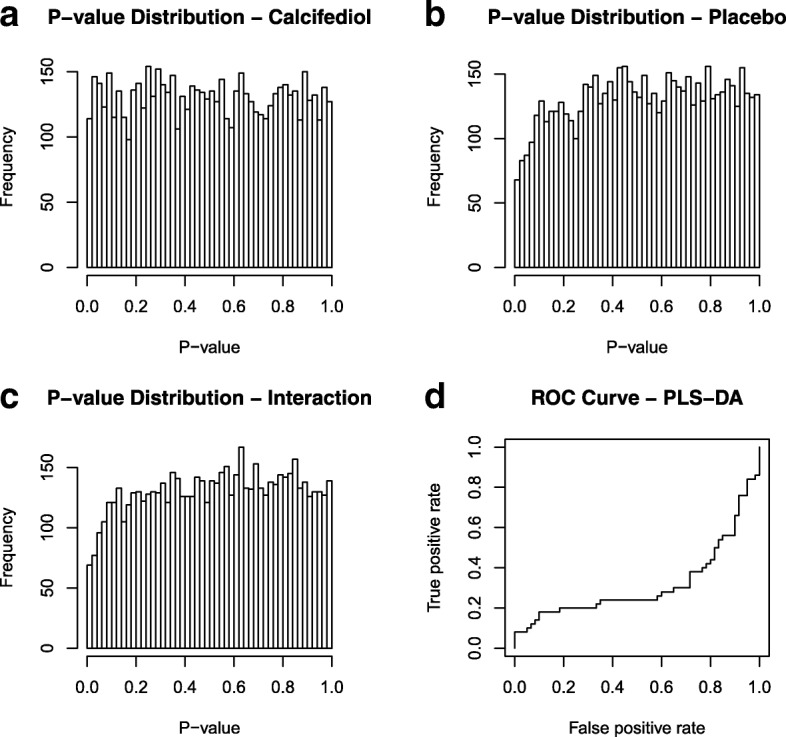
Minimal effect of calcifediol and placebo on skeletal muscle gene expression in vitamin D deficient frail older adults. Top left (**a**): P-values for the change in gene expression for all genes after filtering; before and after calcifediol supplementation. Uniform distribution (i.e. no increased frequency of genes for lower *p*-values) indicates an absence of an effect. Top right (**b**): values for the change in gene expression; before and after in the placebo group. Bottom left (**c**): P-values for the interaction effect (change in the calcifediol group vs. change in placebo group). Bottom right (**d**): receiver operator characteristic curve for the PLS-DA model

**Fig. 4:**
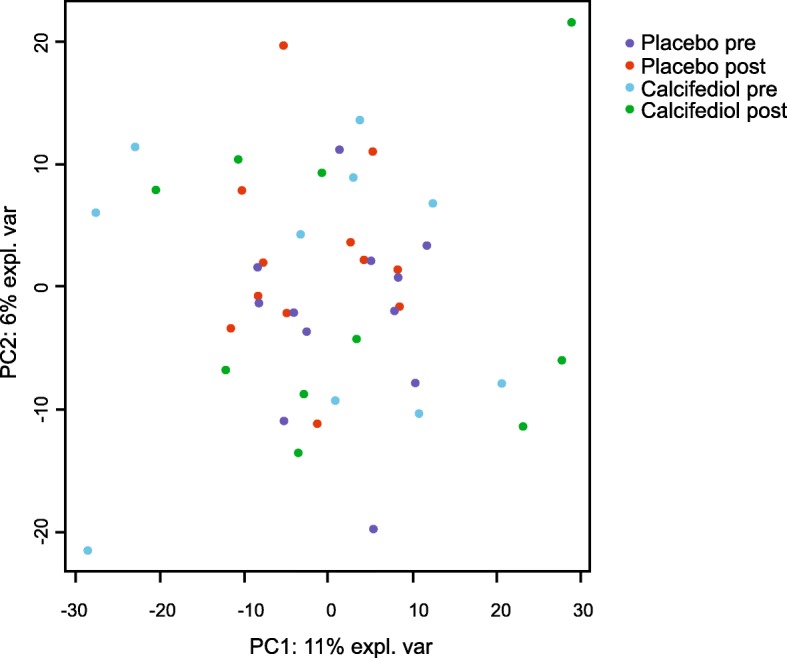
Multilevel principle component analysis (PCA) of gene expression data could not distinguish the four experimental groups. Sample plot from an unsupervised multilevel PCA. In multilevel PCA the paired structure of the data, i.e. measurements performed before and after intervention on the same subject, is taken into account to eliminate the inherent between-subject variation. Results revealed no separation between the experimental groups

Transcriptomics showed minimal effects of calcifediol supplementation and placebo on the skeletal muscle transcriptome. Volcano plot analysis indicated that only a single gene was significantly altered by more than 2-fold in either the calcifediol or placebo group (*P* < 0.001). Figure 2). Volcano plot analysis also indicated that the overall magnitude of gene expression changes were very similar between the calcifediol and placebo group. Only very few genes in the calcifediol group (14 genes) and placebo group (20 genes) met the significance level of IBMT-based *P*-value< 0.001. Using a more lenient P-value cut-off of 0.01, 278 genes were differentially regulated in the placebo group and 174 genes in the calcifediol group, with an overlap of 6 genes. Unadjusted *P*-values were uniformly distributed in both treatment groups and for the interaction between group and time (Fig. 3 a, b, c). Accordingly, genes with a low P-value are likely to be false positives. Correcting for multiple testing using false discovery rate led to no differentially expressed genes using any sensible cut-offs (all q-values ~ 1). Using PLS-DA we attempted to separate the transcriptomic response to calcifediol supplementation from the response in the placebo group. This approach did not reveal any consistent patterns in the data (AUROC < 0.5 during 5 × 5-fold repeated cross-validation, Fig. 3d; accuracy of 0.46, Kappa of − 0.12). Multilevel principle component analysis indicated that the samples from the four different groups were uniformly distributed, showing no clear clustering (Fig. 4).

**Fig. 5:**
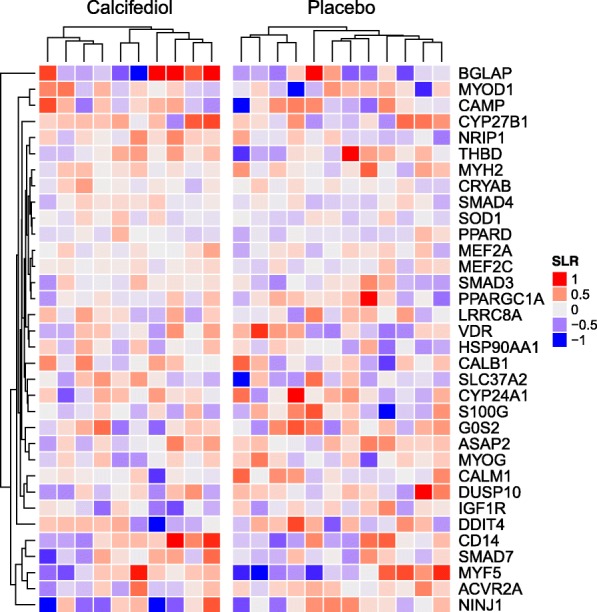
Lack of effect of calcifediol supplementation on expression of putative VDR-dependent genes. Heatmap of gene expression changes (signal log ratio, SLR) of putative genomic targets of VDR

Genes previously described as putative target genes for the vitamin D receptor [38–40] did not show differential expression, with the exception of the insulin-like growth factor 1 receptor (IGF1R, *P* < 0.05, fold change of − 1.27 for the interaction effect between time and treatment; Fig. 5).

**Fig. 6:**
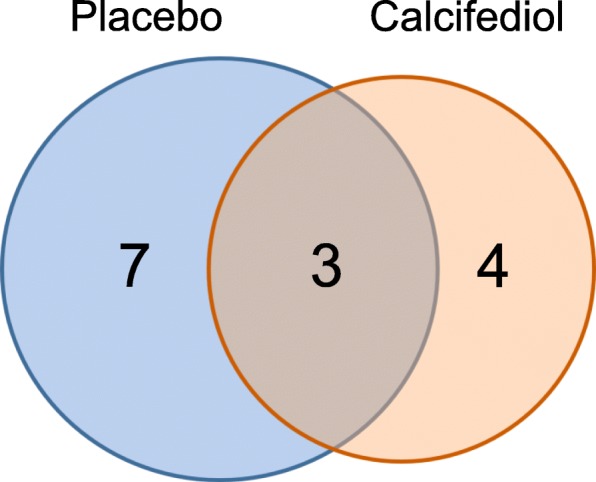
Partially overlapping effect of calcifediol and placebo on muscle transcriptome at the pathway level. Gene set enrichment analysis was performed separately for the effect of calcifediol treatment and placebo treatment. Venn diagram showing overlap in upregulated gene sets (FDR q-value< 0.05) by placebo and calcifediol. The overlapping gene sets are: WP2798.ASSEMBLY. OF.COLLAGEN.FIBRILS.AND.OTHER.MULTIMERIC.STRUCTURES, COLLAGEN. FORMATION, and ASSEMBLY.OF.COLLAGEN.FIBRILS.AND.OTHER.MULTIMERIC. STRUCTURES

Finally, the effects of calcifediol and placebo on specific pathways was investigated. Gene set enrichment analysis yielded 7 significant positively enriched (false discovery rate / FDR q-value < 0.05) genesets in the calcifediol group and 10 significant positively enriched genesets in the placebo group, with 3 overlapping genesets (Fig. 6). The three overlapping genesets were all related to collagen fibrils. These data indicate a time effect on the collagen pathway, which is independent of the type of treatment. Gene set enrichment analysis did not yield any negatively enriched genesets (FDR q-value < 0.05).

## Discussion

This study was initiated on the basis of the assumption that vitamin D supplementation would be able to elicit changes in gene expression in skeletal muscle of vitamin D deficient pre-frail older adults. Contrary to our expectation, the effect of vitamin D on the muscle transcriptome was very weak to non-existent. Although supplementation with calcifediol led to a significant increase in vitamin D status as determined by total 25(OH)D levels (34.1 ± 9.3 to 87.3 ± 20.6, *p* < 0.001), we were unable to confidently identify genes that were affected by calcifediol supplementation. Neither a univariate technique (Limma) nor a multivariate technique (PLS-DA) led to the identification of a robust signature of vitamin D supplementation in skeletal muscle. This is also in accordance with the lack of effect on muscle function [41].

Using a very lenient cut-off we found 174 genes differentially regulated in the calcifediol supplemented arm. However, the same cut-off yielded 278 differentially regulated genes in the placebo group. Interaction contrast for time x treatment led to identification of 190 genes. Given the flat *p*-value distributions, it is difficult to attribute these differentially expressed genes to vitamin D supplementation without risking an unacceptable number of false positives.

Known VDR target genes were not significantly affected by calcifediol treatment. Many VDR targets were identified in immune cells, particularly via microarray studies using immune cells [39]. Chromatin immunoprecipitation sequencing (ChIP-seq) data suggests that the target genes for VDR can vary greatly depending on the cell type [42]. To our knowledge, no ChIP-seq analysis has been carried out on skeletal muscle cells. Other genes were selected based on a recent paper by Hassan-Smith and colleagues [40], in which muscle gene expression was correlated with circulating levels of 1,25(OH_2_)D_3_ and 25(OH)D. Correlations were statistically significant but nevertheless generally weak (~ 0.3–0.5).

There are several possible explanations as to why vitamin D supplementation failed to alter gene expression in our study. Importantly, there is still major discussion on whether VDR is actually expressed in human skeletal muscle. Reports of VDR expression in skeletal muscle go back several decades [43, 44]. However, more recently it was revealed that antibodies against VDR may not be sufficiently specific, leading to overestimation of VDR protein levels in skeletal muscle [20]. A later study detected VDR protein expression in mouse skeletal muscle using the same VDR D6 antibody but using a different western blotting protocol [38]. In this study, 1,25(OH)_2_D_3_ also caused a dose-dependent induction of the VDR target gene CYP24A1 in C2C12 and primary myotubes. In our study, we did not observe any change in cytochrome P450 family 24 subfamily A member 1 (CYP24A1) expression upon calcifediol supplementation. Ceglia et al. also observed VDR in human skeletal muscle, again with the same highly specific VDR D6 antibody, and observed that VDR is primarily expressed in myonuclei [45]. Olsson and colleagues observed that whereas human muscle precursor cells and cultured myotubes express ample amounts of VDR, mature human skeletal muscle does not [17].

The results of Olson and colleagues are in line with our observations. The exclusive presence of VDR in proliferating satellite cells might indicate that VDR is only important in muscle at the developmental stage or after muscle tissue injury. During muscle injury, the normally quiescent satellite cells are activated and turn into proliferating muscle precursor cells. Hence, it is conceivable that in adults vitamin D supplementation may only affect gene expression after muscle injury. The potential role of vitamin D in muscle injury response suggests that vitamin D may have a role in the adaptive response to exercise training.

Another possibility is that the participants in this study were not sufficiently deficient to observe an effect. We did not include individuals with a vitamin D status below 20 nmol/L due to ethical concerns. It is conceivable that very severe deficiency has a much stronger impact on muscle function, thus leading to a bigger observable effect. However, severe deficiency might also lead to alterations in calcium and phosphate metabolism, each of which can affect muscle function [46, 47].

Our study has limitations. First of all, subjects were supplemented with calcifediol for 6 months. It can be hypothesized that the effects of calcifediol on skeletal muscle gene expression may be more pronounced after short term supplementation. It is possible that long term supplementation leads to a new steady state. Second, only a subset of subjects in the calcifediol trial were used for the transcriptomics analysis. However, there are no indications that our study was underpowered, or that a specific bias was introduced between the placebo and calcifediol groups. Finally, we cannot rule out that the lack of discernible effect of calcifediol on skeletal muscle gene expression is due to a low signal to noise ratio, although we consider this explanation unlikely.

## Conclusion

Calcifediol supplementation did not significantly influence the skeletal muscle transcriptome among frail older adults in this randomized, double-blind placebo-controlled clinical trial. Our findings indicate that the effects of vitamin D supplementation on the skeletal muscle transcriptome may be either absent, weak, or limited to a subset of muscle cells.

## Abbreviations

1,25(OH2)D3: Calcitriol; 1,25-dihidroxyvitamin D
24,25(OH2)D3: 24,25-dihidroxyvitamin D
25(OH)D3: Calcifediol; 25-hydroxyvitamin D
AUROC: Area Under Receiver Operator Characteristic
ChIP-seq: Chromatin Immunoprecipitation sequencing
CYP24A1: Cytochrome P450 family 24 subfamily A member 1
FDR: False Discovery Rate
IGF1R: Insulin-like Growth Factor 1
NUSE: Normalized Unscaled Standard Error
PLS-DA: Partial Least Squares Discriminant Analysis
RMA: Robust Multiarray Average
ROC: Receiver Operator Characteristic
UPC: Universal ExPression Codes
VDR: Vitamin D Receptor
